# Insulin resistance by the triglyceride-glucose index in a rural Brazilian population

**DOI:** 10.20945/2359-3997000000509

**Published:** 2022-08-04

**Authors:** Júlia Rabelo Santos Ferreira, Eliana Zandonade, Olívia Maria de Paula Alves Bezerra, Luciane Bresciani Salaroli

**Affiliations:** 1 Universidade Federal do Espírito Santo Programa de Pós-graduação em Saúde Pública Vitória ES Brasil Programa de Pós-graduação em Saúde Pública, Universidade Federal do Espírito Santo (UFES), Vitória, ES, Brasil; 2 Universidade Federal de Ouro Preto Escola de Nutrição Departamento de Nutrição Clínica e Social Ouro Preto MG Brasil Departamento de Nutrição Clínica e Social, Escola de Nutrição, Universidade Federal de Ouro Preto (UFOP), Ouro Preto, MG, Brasil; 3 Universidade Federal do Espírito Santo Programa de Pós-graduação em Nutrição e Saúde Vitória ES Brasil Programa de Pós-graduação em Nutrição e Saúde, Universidade Federal do Espírito Santo (UFES), Vitória, ES, Brasil

**Keywords:** Insulin resistance, TyG index, rural populations

## Abstract

**Objective:**

The aim of this study was to estimate the prevalence of insulin resistance (IR) in a rural population in Brazil, to verify its association with sociodemographic, labor, lifestyle, and health factors.

**Subjects and methods:**

This is a cross-sectional study with 790 farmers in the state of Espírito Santo/Brazil. Triglyceride-glucose (TyG) was calculated and a cut-off point of Ln 4.52 was used. A hierarchical logistic regression for the association of insulin resistance with sociodemographic, labor, lifestyle and health variables of farmers living in Espírito Santo was performed.

**Results:**

The prevalence of insulin resistance was 33.7% (n = 266), and the association with insulin resistance was found in the age group 31 to 40 years of age (OR = 1.85; 95% CI 1.19-2.87); in smokers or former smokers (OR = 1.63; 95% CI 1.08-2.48) and overweight (OR = 3.06; 95% CI 2.22-4.23).

**Conclusion:**

The prevalence of insulin resistance was high in a rural population of Brazil, and was mainly associated with age, smoking and obesity. The use of TyG as an instrument for assessing the health of individuals living in areas where access to health services is difficult, such as rural areas, can represent an important advance in terms of health promotion, protection and recovery. In addition, by identifying the risk factors associated with IR, as well as their consequences, a more adequate scheme for the prevention and treatment of these comorbidities can be defined.

## INTRODUCTION

Insulin resistance (IR) is defined as a metabolic, genetic or acquired dysfunction, in which physiological concentrations of insulin cause a subnormal response in the uptake of glucose by cells, especially muscle and fat ( 1 ). This condition is associated with a series of metabolic abnormalities, such as glucose intolerance, dyslipidemia, hemodynamic changes and inflammation markers, which can lead to the development of chronic diseases such as type 2 diabetes mellitus (DM2), cardiovascular diseases (CVD), arterial hypertension, among others ( 2 ).

The gold standard method for diagnosing IR is the hyperinsulinemic euglycemic clamp ( 3 ), but as it is an expensive and invasive test, it is not available in most laboratories in rural areas. In this context, researchers developed the triglyceride-glucose index (TyG)( 4 ) as a substitute marker that uses simple, routine measures to determine IR. Recently, Brazilian researchers defined a cut-off point for the TyG for the rural population, making it possible and practical to assess their health conditions ( 5 ).

TyG has already been found to have high sensitivity and specificity compared to the hyperinsulinemic euglycemic clamp ( 6 ) and achieved better performance compared to Homeostases Model Assessment-Insulin Resistance (HOMA-IR) when identifying patients with IR ( 7 ). In addition, studies have shown that TyG has been associated with several comorbidities, such as CVD ( 8 ), arterial hypertension ( 9 ), and DM2 ( 10 ).

The literature still lacks studies determining the prevalence of IR in the Brazilian population, being more common those that evaluate the metabolic syndrome (MS) and/or the conditions resulting from IR, such as DM2. In rural populations, this is even more scarce. Until then, a study found a prevalence of 24.2% of IR in two rural communities in the state of Minas Gerais( 11 ), using HOMA-IR. There are still no studies in Brazil that use TyG as a screening tool for IR in rural populations.

Considering this gap in the literature, the importance of studying the health conditions of rural populations, and the new methodology proposed with the use of TyG, the objective of this article was to estimate the prevalence of IR and to verify the association between TyG and sociodemographic, labor, lifestyle and health conditions in a population of rural workers in Espírito Santo – Brazil.

## SUBJECTS AND METHODS

### Study population and design

Cross-sectional, analytical, observational and epidemiological study conducted in the municipality of Santa Maria de Jetibá, in the mountain region of Espírito Santo, Brazil. This article is derived from the project financed by the Research Program of the Unified Health System (PPSUS), through the notice Fapes/CNPq/Decit-SCTIE-MS/SESA nº 05/2015 – PPSUS, entitled “Health conditions and associated factors: a study on farmers in Espírito Santo”. The study was approved by the Research Ethics Committee of the Health Sciences Center of the Federal University of Espírito Santo (UFES), under number 1,856,331 (CAAE 52839116.3.0000.5060). All participants signed the Free and Informed Consent Form (ICF).

The original study involved a representative sample of farmers of both sexes who met the following inclusion criteria: being aged 18 to 59 years old, not pregnant, having agriculture as the main source of income, and having been in full employment for at least six months.

### Sample size calculation

To identify eligible farmers in the original study, data available from individual and family medical records, collected by the Family Health Strategy teams, were used to cover 100% of the 11 health regions in the municipality. Through this research, 7,287 farmers were identified in a total of 4,018 families. The calculation of sample size for the original project was performed considering 50% of prevalence of outcomes (to maximize the sample), 3.5% of sampling error and 95% significance level, finding a minimum sample of 708 producers. 806 farmers were asked to compensate for possible losses. All sample size calculations were performed using the EPIDAT program (version 3.1). Participants were selected by stratified lot, considering the number of families by health region and by Community Health Agent (CHA), in order to respect the proportionality between the 11 regions and between the 80 CHA. Only one individual per family was admitted, avoiding the interdependence of information. In case of refusal or no-show, a new participant was summoned from the reserve list, respecting the sex and the health unit of origin of the person who gave up/refused.

It is noteworthy that, due to the characteristics of the investigated municipality in which family farming predominates, the farmers participating in this study had agricultural practices characterized by the predominance of polyculture and a low degree of mechanization.

For the analyses proposed in this study, the sample size calculation considered an IR prevalence of 20% ( 12 , 13 ), a sample error of 2.5%, and a significance level of 95%, resulting in a minimum sample of 790 farmers.

### Data collection

Data collection from the original study took place between December 2016 and April 2017 on the premises of the city’s health units. A semi-structured questionnaire was applied, containing questions about socioeconomic, demographic and occupational characteristics, occupational contact with pesticides, lifestyle, eating habits and health status, including the presence of chronic diseases and self-perceived health. All of this information was obtained through self-report. Anthropometric measurements were also collected, such as waist circumference, hemodynamic data, such as systolic blood pressure (SBP), diastolic blood pressure (DBP) and blood collected for biochemical tests for markers such as thyroid stimulating hormone and total cholesterol and fractions. To obtain biochemical data, 10 mL of blood were collected by venipuncture, after a 12-hour fast. The use of drugs (such as anti-hypertensive, anti-dyslipidemic and anti-diabetic drugs) was used to classify the individual as having the condition for which the drug was intended.

Only the variables of interest for this article were selected.

### Variables selected for this study

The socioeconomic variables used in this study are: sex (male and female), age group (categorized from the age of 30 by decades of life), marital status, education (number of years of study reported by the farmer), ethnicity (whites and non-whites) and socioeconomic class. The socioeconomic classification was determined based on the Brazilian Economic Classification criteria of the Brazilian Association of Research Companies ( 14 ), where A and B are the highest economic levels, C is intermediate, and D or E are low economic levels.

Occupational data included type of production, weekly working hours, and land tenure. The variables related to lifestyle were physical activity, in addition to the activity performed in the field (categorized as “yes, more than 150 minutes per week”; “yes, less than 150 minutes per week”; “no”), smoking and alcohol consumption, all obtained by self-report. A “smoker” was considered to be a farmer who reported smoking; an “ex-smoker” is someone who did not smoke, but who had smoked in the past; a “non-smoker” was a farmer who reported never having smoked. Alcohol intake was assessed by asking, “How often do you drink alcohol?” Farmers who reported consuming alcohol, regardless of time or quantity, were categorized as “Consumers”. Those who reported not consuming alcoholic beverages were classified as “Do not consume”. Farmers were also asked whether they performed any other physical activity in addition to those related to agricultural work. The answers were categorized as “Yes” or “No”, regardless of the type, time or intensity of the exercise performed.

Anthropometric data were collected according to standard procedures. Weight was measured using a digital scale Omron-514C^®^, with a capacity of 150 kg and precision of 0.1 kg. Three non-consecutive measurements were made, the first being discarded and the average of the last two considered as the final measurement. Height was measured with the Sanny portable stadiometer model ES-2060^®^, to the nearest 0.1 mm. The body mass index (BMI) was calculated by dividing the weight at height squared, categorized according to the WHO cutoff points and regrouped in “Low Weight/Eutrophy” when BMI ≤ 24.9 kg/m^2^ and “Overweight/Obesity” when BMI > 24.9 kg/m^2^. The TyG index was calculated from the equation: = Ln [fasting triglycerides (mg/dL) x fasting glycemia (mg/dL)]/2. The TyG index is expressed on a logarithmic scale ( 4 ). In this study, the cutoff point used for the diagnosis of IR was Ln 4.52 ( 5 ).

**Figure 1:**
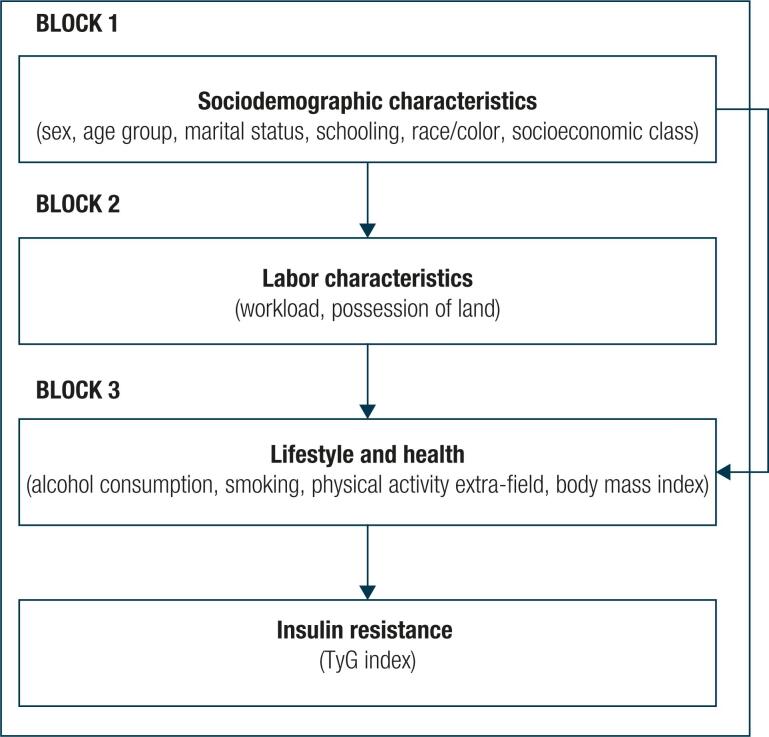
Hierarchical theoretical model of the possible relationships between sociodemographic, work, lifestyle and health variables and the insulin resistance of rural workers – Santa Maria de Jetibá, Espírito Santo, Brazil, 2016-2017

Figure 1 shows the hierarchical theoretical model of the possible relationships between sociodemographic, work, lifestyle and health variables and the insulin resistance of rural workers.

### Statistical analysis

The normality of the variables was tested using the Kolmogorov-Smirnov test. To describe the study variables, percentage measures were used for categorical variables. In order to verify if there was a difference in the proportions between the independent variables and the outcome, Pearson’s chi-square test (X^2^) was used for qualitative variables. When the expected values in the table cells were less than five or when the sum of the column value was less than twenty, Fisher’s exact test was used.

A hierarchical logistic regression for the association of insulin resistance with the independent variables and the TyG Index was performed, including the variables that presented p-value ≤ 0.10 in bivariate analysis. In the first model, only sociodemographic variables were included; to the second model, variables related to work were added; in the third model, lifestyle and health variables were added. The final model was performed using the Forward LR method, in which the weekly working hours variable was removed by the mathematical model itself. For all of them, the assumptions of absence of multicollinearity (tolerance > 0.1 and variance inflation factor < 10) were tested, minimum sample size for the number of variables in the model (> 20 individuals per variable in the model and > 5 cases in each category of variables) and absence of outliers.

Statistical analyzes were performed using the statistical program IBM SPSS Statistics 23 (Armonk, NY: IBM Corp), with a significance level of α < 5%.

### Ethics

The study was approved by the Research Ethics Committee (CEP) of the Health Sciences Center (CCS) of the Federal University of Espírito Santo (UFES), under protocol number 1,856,331. All patients signed the Free and Informed Consent Form (ICF).

## RESULTS

The sociodemographic, labor and health characteristics of the sample can be seen in Table 1 . Of the 806 farmers invited to participate in the survey, 790 individuals completed the survey. Of these, 267 were insulin-resistant (33.8%). Table 2 shows the bivariate analysis between the independent variables and TyG. There is a difference between the proportions in the categories of age group (p < 0.001), workload (p = 0.015), smoking (p = 0.010) and body mass index (p < 0.001).

**Table 1:** General characteristics of study population

Variables		n	%
Sociodemographic		413	52.3
Sex	Male	377	47.7
	Female		
Age group	≤30 years	213	27.0
	31 to 40 years	231	29.2
	41 to 50 years	195	24.7
	>50	151	19.1
Marital status	Single	59	7.5
	Married/living with a partner	678	85.8
	Divorced/separated/widowed	53	6.7
Schooling	>4 years	533	67.5
	4 to 8 years	173	21.9
	>8 years	84	10.6
Race/color	White	702	88.9
	Non-white	88	11.1
Socioeconomic class	A or B	58	7.3
	C	395	50.0
	D or E	337	42.7
Labor			
Workload	≤40 hours/week	162	20.5
	>40 hours/week	628	79.5
Possession of land	Owner	609	77.1
	Non-owner	181	22.9
Health			
Triglycerides	Normal	645	81.6
	High	145	18.4
Glucose	Normal	757	95.8
	High	33	4.2
Total cholesterol	Normal	430	54.5
	High	359	45.5
Blood pressure	Normal	244	30.9
	Prehypertension	263	33.3
	Hypertension	283	35.8

n = 790. Different values mean data loss.

**Table 2:** Bivariate analysis between the TyG index and sociodemographic, labor, health and lifestyle variables

Variables		TyG index			
		Normal	High	Total	p-value
		(≤4.52) a	(>4.52) b	n = 790	
		n (%)	n (%)	n (%)	
Sex	Male	278 (53.1)	135 (50.8)	413 (52.3)	0.547 *
	Female	246 (46.9)	131 (49.2)	377 (47.7)	
Age group	≤30 years	161 (30.7)	52 (19.5)	213 (27.0)	**<0.001**
	31 to 40 years	162 (30.9)	69 (25.9)	231 (29.2)	
	41 to 50 years	112 (21.4)	83 (31.2)	195 (24.7)	
	>50	89 (17.0)	62 (23.3)	151 (19.1)	
Marital status	Single	37 (7.1)	22 (8.3)	59 (7.5)	0.597
	Married/living with a partner	449 (85.7)	229 (86.1)	678 (85.8)	
	Divorced/separated/widowed	38 (7.3)	15 (5.6)	53 (6.7)	
Schooling	>4 years	345 (65.8)	188 (70.7)	533 (67.5)	0.073
	4 to 8 years	127 (24.2)	46 (17.3)	173 (21.9)	
	>8 years	52 (9.9)	32 (12.0)	84 (10.6)	
Race/color	White	468 (89.3)	234 (88.0)	702 (88.9)	0.632 *
	Non-white	56 (10.7)	32 (12.0)	88 (11.1)	
Socioeconomic class	A or B	46 (8.8)	12 (4.5)	58 (7.3)	0.078
	C	254 (48.5)	141 (53.0)	395 (50.0)	
	D or E	224 (42.7)	113 (42.5)	337 (42.7)	
Workload	≤40 hours/week	94 (17.9)	68 (25.6)	162 (20.5)	0.015*
	>40 hours/week	430 (82.1)	198 (74.4)	628 (79.5)	
Possession of land	Owner	405 (77.3)	204 (76.7)	609 (77.1)	0.858 *
	Non-owner	119 (22.7)	62 (23.3)	181 (22.9)	
Physical activity extra-field	No	426 (81.3)	219 (82.3)	645 (81.6)	0.771 *
	Yes	98 (18.7)	47 (17.7)	145 (18.4)	
Alcohol consumption	Non-drinking	290 (55.3)	153 (57.5)	443 (56.1)	0.596 *
	Drinking	234 (44.7)	113 (42.5)	347 (43.9)	
Smoking	Non-smoker	454 (86.6)	211 (79.3)	665 (84.2)	0.010*
	Current and past smoker	70 (13.4)	55 (20.7)	125 (15.8)	
Body mass index	Low weight/eutrophy	306 (58.4)	81 (30.5)	387 (49.0)	<0,001*
	Overweight/obesity	218 (41.6)	185 (69.5)	403 (51.0)	

Pearson’s chi-square test.

* Fischer’s exact test. In bold: statistically significant values.

a n = 524;

b n = 266.

Table 3 shows the crude and adjusted values in accordance to 4 hierarchical models, according to the classes of variables that showed statistical difference in the bivariate analysis. It is observed that all categories (with the exception of the age group from 31 to 40 years old) were associated with TyG in the crude model. However, some associations did not hold as the model was adjusted. In model 3, which contains all the variables, the age group from 41 to 50 years (p = 0.006), smoking (p = 0.020) and being overweight (p <0.001) were shown to be risk factors for IR. Finally, using the forward LR variable selection method, the labor variable workload was removed from the model, and it was concluded that there is an increase of almost two times in the chance of the farmer between 41 and 50 years of age to present IR, compared to individuals younger (OR = 1.85; 95% CI 1.19-2.87); 1.63-fold increase in the risk of IR for smokers (95% CI 1.08-2.48) and approximately three-fold for overweight farmers (95% CI 2.22-4.23).

**Table 3:** Hierarchical logistic regression for the association of insulin resistance with sociodemographic, labor, lifestyle and health variables of farmers living in Espírito Santo

Variables		p-value	OR Crude (CI 95%)	p-value	Model 1 (CI 95%)	p-value	Model 2 (CI 95%)	p-value	Model 3 (CI 95%)	p-value	Model 4 (CI 95%)
Age group											
	≤30 years		1		1		1		1		1
	31 to 40 years	0.198	1.31 (0.87-2.01)	0.198	1.32 (0.87-2.01)	0.202	1.32 (0.86-2.01)	0.663	1.10 (0.71-1.71)	0.664	1.10 (0.71-1.71)
	41 to 50 years	**<0,001**	2.29 (1.50-3.50)	**<0.001**	2.29 (1.50-3.50)	**<0.001**	2.29 (1.50-3.49)	**0.006**	1.85 (1.19-2.87)	**0.006**	1.85 (1.19-2.87)
	>50	**0,001**	2.16 (1.38-3.38)	**0.001**	2.16 (1.38-3.38)	**0.002**	2.06 (1.31-3.24)	0.216	1.36 (0.84-2.21)	0.163	1.41 (0.87-2.29)
Workload											
	≤40 hours/week		1				1		1		
	>40 hours/week	**0.013**	0.64 (0.45-0.91)			**0.027**	0.67 (0.46-0.95)	0.081	0.72 (0.49-1.04)		
Smoking											
	Non-smoker		1						1		1
	Current and past smoker	**0.008**	1.69 (1.15-2.50)					**0.020**	1.64 (1.08-2.49)	**0.021**	1.63 (1.08-2.48)
Body mass index											
	Low weight/eutrophy		1						1		1
	Overweight/obesity	**<0,001**	3.21 (2.34-4.39)					**<0.001**	3.01 (2.18-4.16)	**<0.001**	3.06 (2.22-4.23)

In bold: statistically significant values (p < 0.05). Caption: OR: odds ratio; CI: confidence interval. Model 1: sociodemographic variables; Model 2: sociodemographic and labor variables; Model 3: sociodemographic, labor, lifestyle and health variables Model 4: final model, using the Forward LR variable selection method.

## DISCUSSION

In Brazil, this is one of the first articles to assess insulin resistance and its associated factors using TyG as a diagnostic method, and the first in a rural population. The representative sample, stratified and randomly selected, allows extrapolating the results to the target population.

There was a high prevalence of IR (33.8%) in the rural population of Espírito Santo, higher than the state capital (10.4%) ( 12 ), and rural area in Minas Gerais (24.2%) ( 11 ). However, the last two studies use HOMA-IR as a diagnostic parameter for IR, which limits the comparison of results. IR is closely related to cardiovascular risk factors ( 15 ), so the early identification of this metabolic alteration allows for the prevention of diseases and improved quality of life ( 16 ).

This study showed an association of IR with age in both bivariate and multivariate analysis, being more prevalent in the 41 to 50 age group, in line with that found in Vitória ( 12 ). Other researchers found that age was prone to increase in the highest quintiles of TyG ( 8 , 17 ).

An association of TyG with smoking was also found, with those individuals who smoked almost twice as likely to present IR when compared to those who did not smoke. A study of 1,777 participants in China also found an association between TyG and tobacco use among males ( 18 ). Smoking is associated with decreased insulin secretion, HOMA-IR and hyperinsulinemia ( 19 ). Experimental studies have suggested that smoking can cause and worsen IR, mainly by stimulating the release of catecholamines and other insulins in anti-hormones, impairing the intracellular glucose metabolism pathway, causing disturbances in lipid metabolism and increasing vascular endothelial dysfunction ( 20 , 21 ).

In this study, obese individuals were three times more likely to have a high TyG value when compared to eutrophic and low weight. This interaction between TyG and excess weight has also been verified by other authors ( 4 , 18 ). This can be explained by the increased influx of lipids that occurs in obesity, which exceeds the storage capacity of adipose tissue and results in the accumulation of lipids in the muscle and liver ( 22 ). In this situation of hypertriglyceridemia, the action of insulin is blocked by inhibiting binding to its receptor, with a consequent reduction in hepatic glycogen synthesis and decreased glucose uptake by muscle ( 23 ). That is, the use of insulin-stimulated glucose is limited by the increased oxidation of fatty acids ( 24 ). The competition for oxidation and absorption between glucose and fatty acids results in glucose metabolism impaired by the oxidation of fatty acids ( 25 ).

Obese individuals are insulin resistant and have disturbances in the metabolism of lipoproteins, being more pronounced in obese individuals with hypertriglyceridemia ( 26 ). In addition, it has also been reported that the increase in triglyceride levels in individuals with visceral obesity may be attributable to IR, corroborating the importance that triglycerides have in the pathogenesis of IR and the biological plausibility of using TyG as a substitute in the identification of IR ( 4 ).

The modernization of agriculture in Brazil reached its peak with the “Green Revolution” ( 27 ), whose characteristics included the replacement of products acquired from nature by those selected industrially, the massive and indiscriminate use of fertilizers and pesticides, agricultural machinery and equipment ( 28 ). This process of mechanization of agriculture directly affected small rural producers, intensifying rural exodus, increasing the number of rural poor, violence, environmental destruction and crime ( 29 ).

In addition to the situation of instability in the field, the rural worker is exposed to a series of risk factors and damages inherent to the characteristics of the work, such as accidents with hand tools and machines, poisonous animals, exposure to solar radiation for long periods, exposure to noise, pesticides, among others ( 30 ).

Added to this reality is the fact that most rural areas do not have a specialized health service and most doctors are general practitioners, in addition to having fewer health professionals available ( 31 ). Some evidence suggests worse health conditions and a higher prevalence of diseases among rural populations compared to other groups ( 32 , 33 ), in addition to a high prevalence of cardiovascular risk factors, such as excess of weight, arterial hypertension, metabolic syndrome, and smoking ( 11 , 34 , 35 ).

It is important to note that this is the first article using TyG as a discriminator of IR in a rural population in Brazil. Due to its simple and low cost character, its use in clinical practice could represent an advance in screening individuals at risk of developing a series of other pathologies, especially in areas with little access to more expensive and invasive exams.

Some limitations of the study must be considered. First, its cross-sectional character requires greater caution when interpreting the results due to the possibility of reverse causality. In addition, due to the scarcity of articles in the literature using the same methodology in a similar population, the results could not be compared more accurately. Finally, the differences in the calculations of the TyG formula may represent a limitation, since authors use different cutoff points for TyG around the world, ranging from 4 to 9, depending on how the formula was used.

In conclusion, the prevalence of IR according to the high TyG index was high in a rural population in the southeastern region of Brazil, and was mainly associated with age, smoking and obesity. The use of TyG as an instrument for assessing the health of individuals living in areas where access to health services is difficult, such as rural areas, can represent an important advance in terms of health promotion, protection and recovery. In addition, by identifying the risk factors associated with IR, as well as their consequences, a more adequate scheme for the prevention and treatment of these comorbidities can be defined.
